# Incremental cost of premature birth – a public health care payer perspective from Hungary

**DOI:** 10.1186/s12913-023-09697-w

**Published:** 2023-06-24

**Authors:** Gábor Kovács, Zsolt Abonyi-Tóth, Petra Fadgyas-Freyler, Zoltán Kaló

**Affiliations:** 1 Doctoral School of Sociology, Eötvös Loránd University, Budapest, Hungary; 2 Syreon Research Institute, Budapest, Hungary; 3 University of Veterinary Medicine, Budapest, Hungary; 4 RxTarget Ltd, Budapest, Hungary; 5 National Health Insurance Fund Management, Budapest, Hungary; 6 Center for Health Technology Assessment, Semmelweis University, Budapest, Hungary

**Keywords:** Premature infant, Cost of illness, Public payer, Cost modelling

## Abstract

**Background:**

Preterm birth remains a significant burden to families, health systems and societies. The aim was to quantify the incremental prematurity-related public health expenditure in Hungary and to estimate the potential impact of a decrease in the prevalence of prematurity on the public payer’s spending.

**Methods:**

Over a 6-year time horizon, public financing data of inpatient, outpatient and pharmaceutical care for children born at ≥ 25 weeks of gestation in 2009/2010 were retrieved from the Hungarian National Health Insurance Fund database. In descriptive analysis, the public payer’s spending was given as cost/capita. The impact of a decrease in prematurity prevalence was specified as the total budget impact. An exchange rate of 294 Hungarian forint/Euro was applied.

**Results:**

A total of 93,124 children (including 8.6% who were premature babies) were included in the analysis. A strong negative relationship was shown between gestational age and per capita cost. The 6-year cost of care for the cohort born at 26 weeks of gestation (28,470 Euro per capita) was 24 times higher than that for the cohort born at 40 weeks. First-year inpatient spending accounted for the largest proportion of total health care spending across all gestational ages. All investigated prematurity complications (retinopathy of prematurity, necrotizing enterocolitis, bronchopulmonary dysplasia, intraventricular cerebral bleeding and leukomalacia) resulted in additional significant incremental spending. If 70% of pregnancies ending with preterm birth could be prolonged by 1 week, the savings would be almost 7.0 million Euros in the first 6 years of life.

**Conclusion:**

This comprehensive analysis of prematurity-related health care spending confirmed that premature infants have much higher costs for care than those born at term in Hungary. These quantitative outcomes can provide essential inputs for the cost-effectiveness analysis of medical technologies and public health interventions that can decrease the prevalence of premature birth.

**Trial registration:**

Not applicable.

**Supplementary Information:**

The online version contains supplementary material available at 10.1186/s12913-023-09697-w.

## Background

Prematurity, defined as birth before 37 completed weeks (or 259 days) of gestation, [1] is one of the leading morbidity and mortality factors in childhood, although revolutionary developments in the management of premature babies over the past century—including the significant reduction of infectious diseases—have played an important role in decreasing prematurity-related mortality. At the same time, there has been an increasing societal expectation to keep newborns with poorer viability alive. Societal changes have exerted two opposing effects; although improved social and economic status—mainly of women—have decreased the risk of preterm birth, increasing maternal age (partially related to widely accessible fertilization interventions) has a more significant negative impact on the incidence of prematurity [2–4]. Due to improvements in medical technologies and care, an increasing number of preterm infants survive the critical first weeks of life, and the long-term complications of prematurity and their public health implications have become increasingly apparent and require the involvement of a wide range of medical and nonmedical disciplines [5].

Premature babies are born with dysfunction of all organ systems, and consequently, their organs may show permanent damage. Of the complications, retinopathy, neonatal bowel infections, chronic lung disease and brain damage have particular importance in terms of later disabilities and quality of life [6]. In addition, the consequences of prematurity — long-term care in the neonatal intensive care department, anxieties about the infant after their discharge from the hospital, facing prematurity complications, the damage to interpersonal relationships within the family — can also burden the affected families, leading to psychological burdens, decreased quality of life and additional expenses (“parental complications”) [5, 7].

Despite efforts to reduce the prevalence of preterm birth, approximately 10% of births occur before the end of 37 weeks of gestation [8]. The large majority (85%) of premature infants are moderately premature (born at 32–36 weeks of gestation), 11% are very premature (born at 28–31 weeks of gestation) and 4.1% are extremely premature (born before 28 weeks of gestation) [1, 8]. In addition, the prevalence of prematurity showed an increasing trend between 2000 and 2014 in all regions of the world (from 9.8% to 10.6% globally) and in Europe (from 7.0% in 2000 to 8.7% in 2014) [8]. In many European countries, the prevalence increased further between 2014 and 2016. [9].

In Hungary, the annual number of live births between 1995 and 2018 showed a decreasing trend (with some fluctuation), while the number of preterm births was unchanged, so the prevalence of premature births slightly increased from 7.3% at the beginning of the period to the highest prevalence of 9.0% in 2013. The proportion of extremely premature infants—who have the highest risks for adverse outcomes and cause the most expenditure—increased between 1995 and 2000 and then remained constant [10].

Prematurity is a leading mortality factor in the population aged 0 to 5 years, resulting in 17.8% of all deaths in this age range, of which 90% occur in the first month of life [11]. In Hungary, infant mortality considerably decreased between 2004 and 2018 in the whole premature population (from 5.3% to 2.5%), including the extremely, very and moderately premature populations (from 46.5% to 25%, from 10% to 2.9% and from 1.3% to 0.7%, respectively). In turn, the proportion of neonatal mortality within infant mortality did not show changes in this period (approximately 84% in the extremely preterm babies, 73% in the very preterm babies and 56% in the moderately preterm babies, on average) [10].

The burden of a disease on society can be seen as economic burden (cost-of-illness) or the total amount of healthy lives lost (clinical burden)) [12, 13]. Cost-of-illness studies can be categorized by several aspects, such as the types of costs that are taken into account, [14] the stakeholders whose expenditures are analysed, how detailed and comprehensively the services and costs are counted [13, 15–17] and the sources of cost data [18].

A systematic literature review [19]— including 16 studies published after January 1, 2011 —showed that prematurity was consistently associated with considerably higher expenditure for both the short and long term. The incremental expenditure was especially high during the first hospitalization, when extremely premature babies and very and moderately premature babies needed 100-fold and tenfold more inpatient financing, respectively, than term infants [20–23]. Significant additional costs could be demonstrated for the whole first year of life [22–28] regardless of whether the initial hospital costs were included. The additional costs of care for premature babies could also be demonstrated at preschool age [22, 25–27, 29] and thereafter [30]. When analysing the cost of care in the first years of life, the more premature the babies were, the higher the proportion of expenditure paid during the initial hospitalization or in the first year of life [22, 25, 26]. In addition to increased direct health care costs, it was demonstrated that the adults who had been born prematurely earned less than those who were born at term. All studies that quantified the expenditure consequences of the prematurity complications that were investigated in this analysis (i.e., retinopathy of prematurity [ROP], [31–34] necrotizing enterocolitis [NEC], [35–39] bronchopulmonary dysplasia [BPD], [38–42] and intraventricular haemorrhage—periventricular leukomalacia [IVH-PVL] [38, 39, 43]) confirmed that these complications were associated with incremental public health spending.

Any health technologies or policy interventions that aim to reduce the prevalence of prematurity should be assessed by considering expected savings associated with avoided prematurity. The objectives of this study were (1) to quantify incremental prematurity-related public health spending and (2) to estimate how decreases in the prevalence of preterm birth affect public payer’s health spending.

## Methods

This research analysed inpatient, outpatient and pharmaceutical care expenditures covered by the National Health Insurance Fund (NHIF) in the first 6 years of children’s lives. The following inclusion criteria were applied for the study population: (1) children born in Hungary between January 1, 2009, and December 31, 2010; (2) children whose gestational age was equal to or greater than 25 weeks; (3) children whose gestational age could be determined; and (4) children for whom all data for inpatient, outpatient and pharmaceutical care expenditures from birth to 6 years of life were available. The NHIF provided the following data: resource use during the first 6 years of life, the chronological age (in semiannual breakdown) of infants, and whether a patient suffered from one or more predefined prematurity complications (i.e., ROP, NEC, BPD, IVH-PVL). Analysis was performed on patient-level data stored at the NHIF; however, only the aggregated data were provided to the researchers for data privacy reasons, that allowed to calculate only mean costs in the descriptive analysis.

In Hungary, the reimbursement system of acute inpatient care is based on Diagnostic Related Groups (DRGs), and outpatient care is reimbursed based on the German point system. The reimbursement of pharmaceutical care outside hospitals is specified by drug classes and indications.

As the data for gestational age in the DRG records and ICD codes in the public payer’s database were incomplete, gestational age was determined based on the time (weeks) elapsed since the date of a mother’s alpha-fetoprotein screening test (a test that was part of routine pregnancy care in Hungary in 2009 and 2010 and was performed at 17 weeks of gestation) [44]. The analysis was performed by weeks of gestation or gestational age groups (applying the following classification: 25–27 weeks: extremely preterm; 28–31 weeks: very preterm; 32–36 weeks: moderately preterm), [1] chronological age (in semiannual breakdown), the types of health services (i.e., inpatient, outpatient and pharmaceutical care), and prematurity complications. When an inpatient care event spread to the next 6-month period of chronological age, the DRG payment of this event was split into two time-proportionate periods. Similarly, near the end of the observational period at year 6, only the time-proportionate part of the inpatient care payments was considered. Spending in Hungarian forints was converted to Euro with an average exchange rate of 294 HUF/EUR between 2009 and 2016.

In the descriptive analyses, public health expenditure per capita in each subgroup was calculated based on the cumulative expenditure in the given period divided by the number of children in the subgroup. The number of children in a given period was calculated as the average number of children who started and completed the period, assuming a constant mortality rate in the given period. In the first 6-month period, however, correction for a higher neonatal mortality rate was made based on aggregated mortality rates at Day 28 after birth.

The descriptive analysis included the total inpatient, outpatient and pharmaceutical public expenditures by gestational age groups and the mean expenditure per capita in each subgroup for the 6-year observational period, the incremental spending per capita between each subgroup, the distribution of yearly inpatient, outpatient and pharmaceutical expenditures over the 6-year observational period by gestational age, and the incremental expenditure associated with selected prematurity complications. The impact of a potential reduction in the prevalence of prematurity was modelled by assuming that 30%, 50% or 70% of the pregnancies between 25 and 36 weeks of gestation could be prolonged by one week. The modelled spending was calculated for the first 6 months of life and the whole 6-year investigation period. In calculating the total cost of care, the aggregated resource use data (i.e., DRGs, German points) was multiplied by the current unit costs (the payment for one DRG or German point).

The study conformed to the Helsinki Declaration of 1964, and its later amendments, and was approved by the Scientific and Research Ethics Committee of the Medical Research Council (registration code: 38,254–2/2019/EKU).

## Results

### Population

From the birth cohorts of 2009 and 2010, 93 124 infants met the inclusion criteria (study population), of whom 8.6% were preterm infants (including 0.25% who were extremely preterm [*N* = 234], 1.0% who were very preterm [*N* = 938] and 7.3% who were moderately preterm [*N* = 6 801]). The study population included 50% of the infants born alive at ≥ 25 weeks of gestation in 2009 and 2010 in Hungary. Therefore, the study population corresponded to a one-year birth cohort. The proportions of male infants were higher than those of females in all gestational subgroups. A vast majority (99.7%) of the infants survived until the end of the first year of life, and 99.6% survived until the end of their sixth year of life. The 1-year survival rates were 61–76% among the extremely premature babies, 89–95% among the very premature babies and 98–99% among the moderately premature babies. The baseline characteristics of the study population are summarized in Table 1.

**Table 1:** Baseline characteristics of the study population

Gestational age groups (weeks)	Number (proportion) of infants	Proportion of males	Prevalence of prematurity complications				Proportion of surviving infants	
			**ROP**	**NEC**	**BPD**	**IVH-PVL**	**6 months of life**	**6 years of life**
** ≤ 27**	234 (0.25%)	60.7%	48.3%	13,2%	37.2%	47.0%	68.2%	67.1%
**28–31**	938 (1.0%)	55.7%	43.1%	4,5%	11.1%	23.8%	95.0%	94.7%
**32–36**	6 801 (7.3%)	54.9%					99.6%	99.3%
** ≥ 37**	85 151 (91.4%)	51.7%					99.9%	99.8%

### Total public health expenditure

The public payer paid almost 70 million Euro in the first 6 months and 141.5 million Euro in the first 6 years of life for inpatient, outpatient and pharmaceutical care of the study population (see Table 2). The proportions of the spending for premature babies (born at ≤ 36 weeks of gestation), who accounted for 8.6% of the whole population, were 38.3% in the first 6 months and 25.4% in the whole 6-year study period. The incremental cost of care was more pronounced for extremely and very preterm babies (0.25% were extremely preterm newborns [≤ 27 weeks of gestation] who required 5.4% of the total expenditure in the first half year of life and 3.2% in the first 6 years of life; these expenditure proportions were 11.9% and 7.1%, respectively, in the case of the 1.0% who were very preterm newborns [28–31 weeks of gestation]).

**Table 2:** Total public health expenditure in the first 6 months and 6 years of life

**Gestational age groups (weeks)**	**Number (proportion) of infants**	**Total public health expenditure** (% of the total expenditure in the given period), *thousand, Euro*	
		**In the first 6 months of life**	**In the 6 years of life**
** ≤ 27**	234 (0.25%)	3 751 (5.4%)	4 585 (3.2%)
**28–31**	938 (1.0%)	8 313 (11.9%)	9 991 (7.1%)
**32–36**	6 801 (7.3%)	14 690 (21.0%)	21 363 (15.1%)
***premature (*** **≤ ** ***36)***	*7* *973 (8.6%)*	*26 753 (38.3%)*	*35 939 (25.4%)*
** ≥ 37**	85 151 (91.4%)	43 112 (61.7%)	105 548 (74.6%)
**Total**	**93 124**	**69 866**	**141 487**

The values indicate total public payer expenditures

### Total public health expenditure per capita

**Fig. 1:**
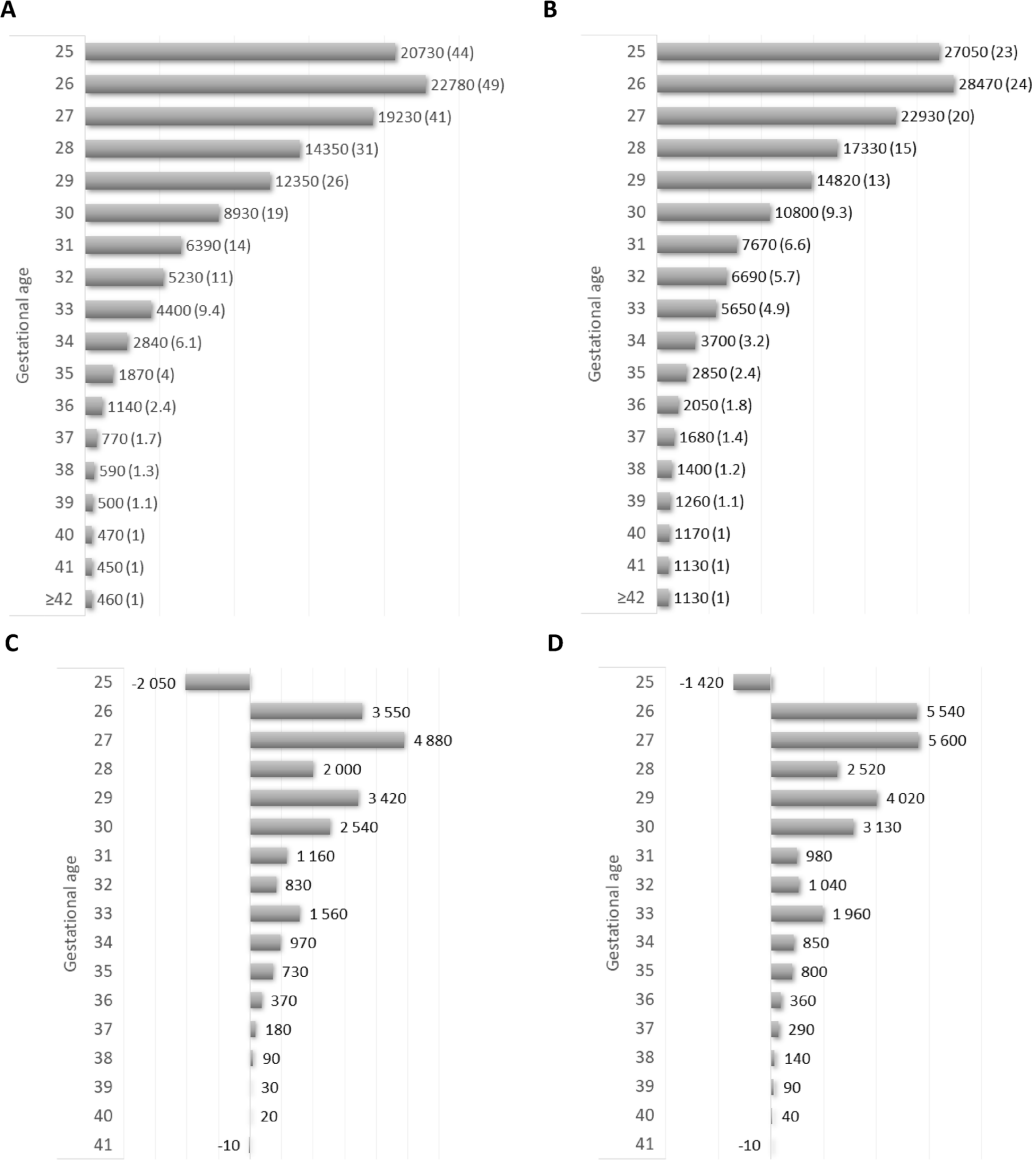
Total expenditure per capita (rounded, Euro) by gestational age (weeks). **A** in the first 6 months, **B** in the first 6 years of life; the values in brackets show how many times higher the costs for a child born at a given gestational age are compared to those of a child born at 40 weeks. **C** The incremental spending in each gestational group compared to the costs of those one week more mature in the first 6 months of life; **D** the incremental spending in the first 6 years of life

Figure 1 shows the total expenditure per capita in the first 6 months (Fig. 1A) and 6 years after birth (Fig. 1B). The values in brackets indicate how many times higher the expenditure paid for a baby of a given gestational age is compared to that of a baby born at 40 weeks of gestation. Both in the first half year and the whole 6-year study period, a strong negative association was detected between gestational age and total expenditure per capita. The care of newborns born at 26 weeks of gestation was the most costly in the first half year and the first 6 years of life; additionally, compared to those born at 40 weeks of gestation, the public health expenditure of these babies was almost 50 times higher in the first half year and 24 times higher in the first 6 years of life. When the incremental spending on children at each gestational age was compared to those born one week later, a decreasing trend was demonstrated (Fig. 1C and D). Interestingly, the costs of newborns born at 25 weeks of gestation were lower than those of newborns born at 26 weeks of gestation in both time horizons: the most acceptable explanation is that much less care was provided for these babies in the first half year of life due to extremely high early neonatal mortality.

### Distribution of the expenditure by* age* and* health care type*

The expenditure per capita by type of health care, weeks of gestation and postnatal age periods are summarized in Table S1, Table S2 and Table S3 (see Supplementary Material). *Inpatient expenditure per capita* showed a decreasing trend with increasing gestational and postnatal age. The strongest association between gestational age and inpatient expenditure could be demonstrated in the first two half years of life (e.g., compared to those born at 40 weeks of gestation, the inpatient expenditure paid for an infant born at 26 weeks of gestation was 57.5 times more in the first half year, while an infant born at 25 weeks of gestation cost 40.5 times more in the second half year). From the second year of life, these multipliers ranged between 1.1 and 6.9. Similar trends could be demonstrated in *outpatient care expenditure per capita*, although the costs for extremely and very premature babies were only 1.8–8.2 times higher in the first two half years of life and 1.5–7.1 times higher between 2 and 6 years of life than those of infants born at 40 weeks of gestation. On the other hand, the *pharmaceutical care expenditure per capita* showed a different trend: while the spending on extremely and very premature infants was only 1.2–4.2 times higher than that of infants born at 40 weeks of gestation in the first two half years, the multipliers between the extremely and very premature versus mature infants were much larger between 2 and 6 years of life (between 2.3 and 21.0).

The relative proportions of yearly inpatient, outpatient and outpatient pharmaceutical care expenditures are shown in Table 3. The first-year inpatient care expenditure represented the highest proportion of the total expenditure paid in the first 6 years of life across all gestational ages. The proportion of the first-year inpatient expenditure showed an association with gestational age, and was over 80% in extremely and very preterm infants and decreased gradually to approximately 40% in mature infants. Although the proportions of expenditure paid in the following years were much smaller than that of the first year, inpatient care dominated the total expenditure across all years.

**Table 3:** Distribution of yearly expenditures in the first 6 years of life by types of care

	Year 1			Year 2			Year 3			Year 4			Year 5			Year 6		
**Gestational age (weeks)**	Inpat	Outpat	Pharm	Inpat	Outpat	Pharm	Inpat	Outpat	Pharm	Inpat	Outpat	Pharm	Inpat	Outpat	Pharm	Inpat	Outpat	Pharm
25	82.4%	0.58%	0.69%	1.4%	0.56%	1.1%	1.7%	0.20%	1.3%	0.42%	0.18%	1.6%	1.3%	0.13%	1.6%	0.91%	0.14%	1.7%
26	82.8%	0.65%	0.82%	1.4%	0.46%	1.3%	0.97%	0.29%	1.5%	0.67%	0.25%	1.6%	0.98%	0.29%	1.5%	0.83%	0.18%	2.1%
27	84.7%	0.76%	0.79%	1.6%	0.53%	1.3%	1.8%	0.31%	0.98%	1.1%	0.29%	0.68%	0.67%	0.24%	0.91%	1.0%	0.23%	0.89%
28	84.0%	1.0%	0.95%	1.4%	0.63%	0.98%	1.1%	0.40%	1.2%	0.93%	0.32%	1.3%	0.81%	0.32%	1.0%	0.72%	0.37%	1.5%
29	84.8%	1.1%	1.2%	1.4%	0.51%	0.90%	1.2%	0.38%	0.82%	1.3%	0.37%	0.90%	0.96%	0.38%	0.99%	0.83%	0.34%	1.0%
30	83.0%	1.4%	1.5%	1.2%	0.65%	1.4%	1.1%	0.36%	1.1%	0.70%	0.48%	1.0%	0.92%	0.30%	1.7%	0.60%	0.32%	1.7%
31	82.8%	1.6%	1.9%	1.6%	0.77%	1.3%	1.2%	0.50%	1.1%	0.92%	0.47%	1.0%	1.1%	0.52%	0.97%	0.62%	0.57%	0.92%
32	78.3%	1.7%	2.2%	3.2%	0.78%	1.2%	1.9%	0.53%	0.85%	1.7%	0.52%	0.89%	1.6%	0.54%	0.98%	1.5%	0.45%	1.2%
33	77.6%	1.9%	2.6%	2.7%	0.99%	1.6%	2.5%	0.60%	1.1%	1.5%	0.59%	0.90%	0.93%	0.71%	0.79%	1.2%	0.73%	0.78%
34	75.9%	2.3%	3.3%	2.8%	0.94%	1.3%	2.2%	0.65%	0.89%	1.8%	0.68%	0.80%	1.9%	0.66%	0.84%	1.4%	0.78%	0.76%
35	66.3%	2.8%	3.9%	4.2%	1.3%	1.8%	3.2%	1.1%	1.3%	2.9%	1.0%	1.2%	2.5%	0.95%	1.2%	2.1%	0.97%	1.2%
36	55.7%	3.1%	4.9%	6.2%	1.5%	2.7%	5.1%	1.2%	1.6%	3.7%	1.2%	1.8%	3.0%	1.3%	1.7%	2.3%	1.3%	1.6%
37	46.1%	3.1%	5.7%	6.8%	1.7%	2.7%	4.9%	1.4%	2.0%	5.7%	1.5%	2.0%	4.8%	1.6%	2.0%	4.4%	1.5%	1.9%
38	41.8%	3.3%	6.5%	8.3%	1.8%	3.2%	5.9%	1.7%	2.5%	4.9%	1.8%	2.5%	3.9%	1.7%	2.4%	3.3%	1.8%	2.5%
39	39.1%	3.3%	7.0%	7.8%	1.8%	3.5%	6.2%	1.6%	2.6%	5.4%	1.8%	2.6%	4.6%	1.8%	2.5%	3.8%	1.9%	2.4%
40	38.5%	3.4%	7.5%	7.8%	1.9%	3.8%	5.8%	1.7%	2.6%	5.5%	1.8%	2.7%	4.3%	1.8%	2.6%	3.8%	1.9%	2.5%
41	38.6%	3.4%	7.8%	7.9%	1.8%	3.8%	5.7%	1.7%	2.6%	5.4%	1.9%	2.5%	4.2%	1.9%	2.5%	3.9%	2.0%	2.3%
≥ 42	38.4%	3.5%	7.8%	7.4%	1.9%	3.8%	5.7%	1.6%	2.8%	5.3%	1.8%	2.6%	4.4%	1.9%	2.5%	4.3%	2.0%	2.2%

*Inpat.* Inpatient care, *Outpat.* Outpatient care, *Pharm.* Pharmaceutical care

### Incremental spending associated with prematurity complications

**Fig. 2:**
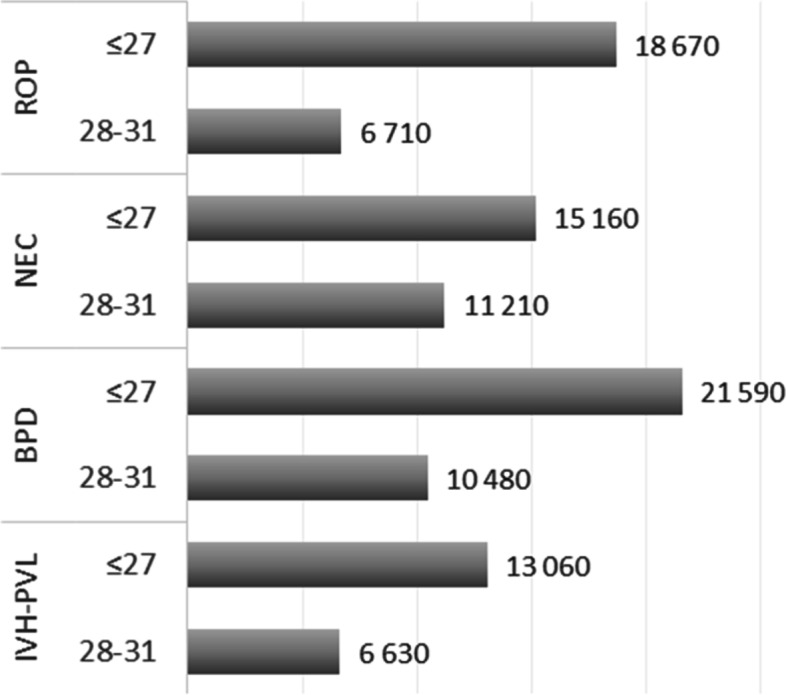
The additional public health expenditure per capita in prematurity-related complications (rounded, Euro). The values show the additional expenditure that the public payer spent in the first 6 years of life for children with the indicated prematurity related complications compared to those without the given complications. The data were provided separately for the extremely and the very preterm populations

When the 6-year total public expenditure per capita was compared in children who were positive versus negative for selected prematurity-related complications, higher values were demonstrated in both extremely and very premature infants across all complications, including ROP, NEC, BDP, and IVH-PVL, as described in Fig. 2. The highest incremental spending was associated with BPD in extremely premature infants.

### Economic implications of reduced prematurity

**Fig. 3:**
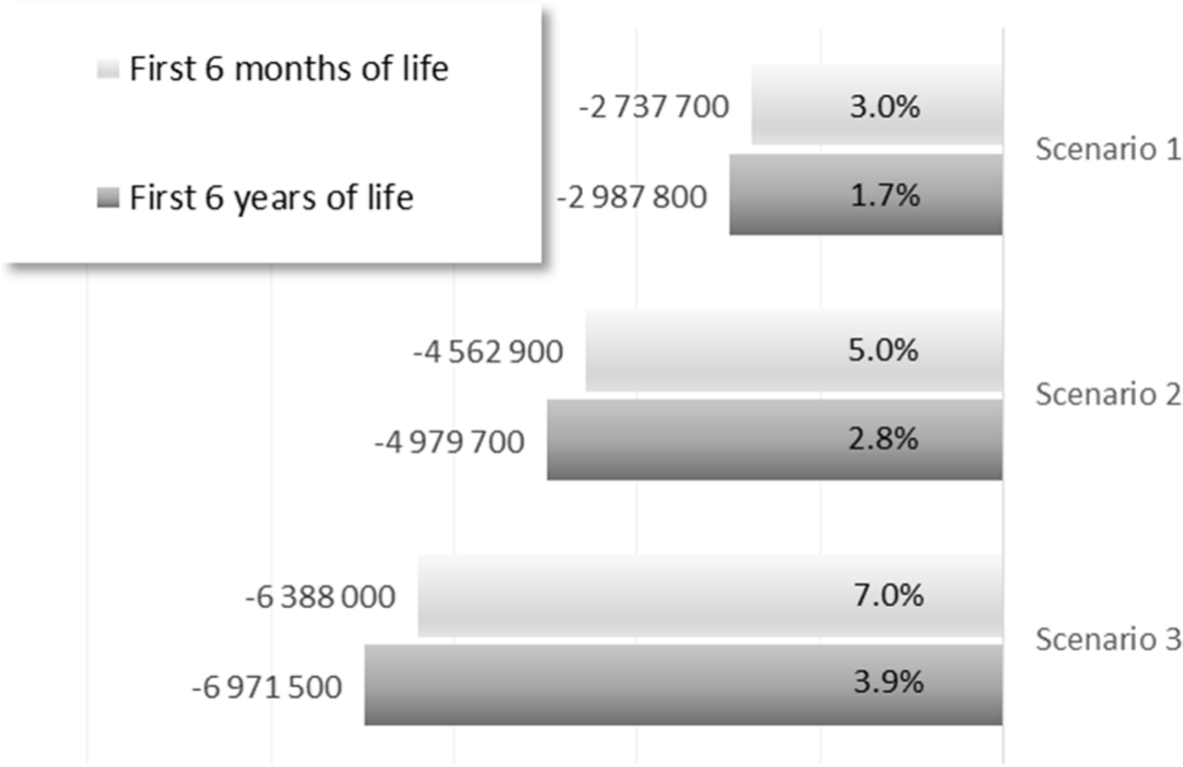
Estimated savings in the first 6 months and the first 6 years of life in absolute values (rounded, Euro) and in proportions of the total public health expenditure related to prolonging gestation by one week in 30% (Scenario 1), 50% (Scenario 2) or 70% (Scenario 3) of pregnancies

The economic impact of a potential reduction in the prevalence of prematurity was modelled in different scenarios by assuming that 30% (Scenario 1), 50% (Scenario 2) or 70% (Scenario 3) of the pregnancies between 25 and 36 weeks of gestation could be prolonged by one week. Public health spending in each scenario was estimated for 6 months and 6 years after birth. Figure 3 shows that the prolongation of 30% of pregnancies by 1 week (Scenario 1) would save more than 2.7 million Euros in the first half year and almost 3 million Euros in the first 6 years, which represent 3.0% and 1.7% of the total budget in the given periods, respectively. If 70% of all pregnancies ending with preterm birth were prolonged by 1 week (Scenario 3), the savings would be 6.4 million Euros in the first half year and almost 7.0 million Euros in the first 6 years of life (representing 7.0% and 3.9% of the budgets in the given periods, respectively).

## Discussion

Prematurity plays an important role in morbidity and mortality in childhood. The health care costs related to prematurity that are covered by both the public payers’ and the families’ medical care are extremely high. Although medical innovation for life-saving interventions in the neonatal period led to significant improvements in survival, it also resulted in an increased prevalence of chronic or even life-long prematurity-related complications. In addition to higher health spending, prematurity-related complications are associated with psychological burdens and decreased quality of life of patients and family caregivers.

Despite all efforts, preterm birth remains a significant problem worldwide (affecting approximately 9–10% of pregnancies). Its prevalence has slightly increased in Hungary in the last two decades, although the large majority of these infants were moderately premature, and the 0–27-day and 0–355-day mortalities have decreased in the last two decades.

Several studies have quantified the association between the severity of prematurity or low birthweight and the health expenditure paid by third-party payers of the affected families. Although these studies were heterogenic in several aspects of their methodologies (time horizon, perspective, cost categories, health system environment, categorization by gestational age or birthweight, etc.), they unanimously confirmed that a younger gestational age at birth or lower birthweight resulted in increased health care costs. It should be emphasized that the systematic review (conducted in March 2018) could not identify any prematurity-related cost analyses from the Central and Eastern European regions.

Our analysis was performed from the perspective of the Hungarian National Health Insurance Fund, as a public payer, and covered public spending on inpatient, outpatient and pharmaceutical care for children born between January 1, 2009, and December 31, 2010. The analysis clearly demonstrated a strong negative relationship between the maturity of infants and expenditure per capita. Although a negative trend was seen between most gestational ages and the type of care, this association was less strong. Premature infants, who represented 8.6% of the newborn population, required almost 40% of the total public spending for all children, and 1.25% of extremely or very premature children required 17.3% of the total public expenditure 6 years after birth. Independent of gestational age, the largest share of the total 6-year expenditure was paid for inpatient care in the first year of life: the share was approximately 40% for mature infants but over 80% for extremely and very preterm infants. By assuming an unchanged health care financing structure (DRG weights, outpatient care financing and reimbursement rates for different drug classes), the prolongation of 30%, 50% and 70% of premature births by 1 week would result in almost 3, 5 and 7 million Euros saved in the first 6 years of life (representing 1.7%, 2.8% and 3.9% or the total public payer budget paid for children in this period). The proportions of savings in the public health care budget would be 3.0%, 5.0% and 7.0% in the first 6 months after birth.

Although the cost per capita in similar studies in the Netherlands, and in the United States [21, 20] was many times higher than that in Hungary, the results are consistent with each other. Marzouk et al.’s analysis by gestational age groups showed a proportional increase in health care spending that was similar to our study [23]. Additionally, similar cost per capita data were provided in the studies that analysed the whole first year of life [22, 25]. It can generally be stated that the ratio of expenditure in each gestational age or age group compared to those born at 40 weeks of gestation was similar in studies from different regions of the world, and the absolute differences are probably due to the differences in the economic status of the countries.

The economic impact of reducing the prevalence of prematurity could be modelled in different ways. The modelling technique chosen in this study used a conservative approach, assuming that a proportion of pregnancies that would have ended with preterm birth would be prolonged by 1 week in 30%, 50% and 70% of cases. The return on investment of health policy interventions (e.g., improved prenatal care) would be highly positive, most likely resulting in cost savings in addition to significant improvements in the survival of children and the quality of life of both children and their family caregivers.

### Limitations

The main limitation of the study is that only a portion of all children born in 2009 and 2010 could be included in the study, as the whole 6-year health care and financing history could be explored for only approximately 50% of the 2009–2010 birth cohort. However, it can be reasonably assumed that the availability of the health care and reimbursement history was independent from the patient characteristics and financing outcomes, so it did not introduce bias in the analysis. An important limitation is that the only aggregated data were provided by the public payer, which prevented researchers from calculating uncertainty measures—such as standard error or any quantiles—in the descriptive analysis and the modelling of costs. On the other hand, due to the large sample size—that was actually equal to the number newborn population born in one year in Hungary—the cost estimations are sufficiently reliable.

Although it is well-known that socioeconomic status of patients influences their health status and related health care costs, the public payer databases did not contain information on socioeconomic status. On the other hand, authors believe that in Hungary there is limited inequity in the access to hospital care for newborn babies in the first 6 months after birth compared with any other services and any other age groups, therefore the most important cost driver in our analysis was not sensitive to socioeconomic status.

Another limitation is that gestational age was determined based on the time elapsed between the date of the mother’s alpha-fetoprotein screening test and the date of birth. Although alpha-fetoprotein screening was strictly specified by local guidelines and enforced before 2012, it might occur in a minority of the cases in which the time of the screening shifted one week forward or backwards.

## Conclusion

As a conclusion, the current study highlighted the significant economic potential of reducing the prevalence of prematurity in Hungary, where the prevalence rate is among the highest in Europe. The calculations may provide essential input for cost-effectiveness analyses of any prenatal care or other policy interventions that aim to reduce the prevalence of prematurity. To the best of the authors' knowledge, this is the first study that comprehensively analysed the incremental costs of prematurity in Central and Eastern Europe.

## Supplementary Information

**Additional file 1.** 

**Additional file 2.** 

**Additional file 3.** 

## Data Availability

The baseline data that used for the analysis have been summarized in Tables in the Supplementary file. The public payer, for data confidentiality reason, do not provide individual demographic, medical and financial data to a researcher, but only cumulative data for pre-specified groups of patients.

## abbreviations

BPD: Bronchopulmonary Dysplasia
DRG: Diagnostic Related Group
IVH: Intraventricular Haemorrhage
NEC: Necrotizing Enterocolitis
NHIF: National Health Insurance Fund
PVL: Periventricular Leukomalacia
ROP: Retinopathy of Prematurity
