# Supplementary material for: Incremental cost of premature birth – a public health care payer perspective from Hungary
Source: BMC Health Serv Res. 2023 Jun 24;23:686. doi: 10.1186/s12913-023-09697-w (PMC10290341; doi:10.1186/s12913-023-09697-w)
Supplement: Supplementary file 1 — Additional file 1. [file 12913_2023_9697_MOESM1_ESM.docx]

**INCREMENTAL COST OF PREMATURE BIRTH – A PUBLIC HEALTH CARE PAYER PERSPECTIVE FROM HUNGARY**

S1. Inpatient expenditure per capita (Euro) by gestational age (weeks) in different periods of life

| **Gest. age (weeks)** | **Periods of life** | | | | | | |
| --- | --- | --- | --- | --- | --- | --- | --- |
|  | **1^st^ half year** | **2^nd^ half year** | **2^nd^ year** | **3^rd^ year** | **4^th^ year** | **5^th^ year** | **6^th^ year** |
| 25 | 20 624 | 2 249 | 369 | 466 | 114 | 338 | 245 |
| 26 | 22 643 | 1 264 | 412 | 275 | 192 | 280 | 237 |
| 27 | 19 095 | 603 | 372 | 410 | 251 | 153 | 230 |
| 28 | 14 202 | 553 | 237 | 189 | 162 | 141 | 124 |
| 29 | 12 188 | 473 | 200 | 175 | 197 | 142 | 123 |
| 30 | 8 775 | 233 | 135 | 118 | 76 | 100 | 65 |
| 31 | 6 252 | 121 | 119 | 92 | 71 | 82 | 48 |
| 32 | 5 090 | 155 | 214 | 125 | 112 | 105 | 98 |
| 33 | 4 266 | 132 | 155 | 144 | 86 | 53 | 66 |
| 34 | 2 718 | 90 | 105 | 82 | 66 | 69 | 53 |
| 35 | 1 762 | 127 | 120 | 92 | 82 | 70 | 59 |
| 36 | 1 047 | 93 | 126 | 105 | 75 | 62 | 46 |
| 37 | 685 | 93 | 115 | 82 | 96 | 81 | 74 |
| 38 | 507 | 77 | 116 | 83 | 68 | 54 | 46 |
| 39 | 424 | 68 | 98 | 79 | 68 | 57 | 48 |
| 40 | 394 | 56 | 91 | 67 | 64 | 51 | 45 |
| 41 | 380 | 56 | 89 | 64 | 61 | 48 | 44 |
| ≥42 | 383 | 52 | 84 | 64 | 60 | 50 | 49 |
