# Supplementary material for: Incremental cost of premature birth – a public health care payer perspective from Hungary
Source: BMC Health Serv Res. 2023 Jun 24;23:686. doi: 10.1186/s12913-023-09697-w (PMC10290341; doi:10.1186/s12913-023-09697-w)
Supplement: Supplementary file 2 — Additional file 2. [file 12913_2023_9697_MOESM2_ESM.docx]

**INCREMENTAL COST OF PREMATURE BIRTH – A PUBLIC HEALTH CARE PAYER PERSPECTIVE FROM HUNGARY**

S2. Outpatient expenditure per capita (Euro) by gestational age (weeks) in different periods of life

| **Gest. age (weeks)** | **Periods of life** | | | | | | |
| --- | --- | --- | --- | --- | --- | --- | --- |
|  | **1^st^ half year** | **2^nd^ half year** | **2^nd^ year** | **3^rd^ year** | **4^th^ year** | **5^th^ year** | **6^th^ year** |
| 25 | 46 | 112 | 153 | 53 | 48 | 35 | 38 |
| 26 | 73 | 113 | 131 | 83 | 71 | 83 | 51 |
| 27 | 69 | 106 | 122 | 70 | 66 | 56 | 52 |
| 28 | 77 | 103 | 109 | 68 | 56 | 56 | 64 |
| 29 | 79 | 80 | 76 | 56 | 54 | 56 | 51 |
| 30 | 77 | 75 | 70 | 39 | 52 | 32 | 35 |
| 31 | 64 | 56 | 59 | 38 | 36 | 40 | 44 |
| 32 | 65 | 47 | 52 | 35 | 34 | 36 | 30 |
| 33 | 59 | 47 | 56 | 34 | 33 | 40 | 41 |
| 34 | 54 | 32 | 35 | 24 | 25 | 25 | 29 |
| 35 | 47 | 32 | 38 | 31 | 29 | 27 | 28 |
| 36 | 40 | 24 | 30 | 25 | 25 | 27 | 27 |
| 37 | 33 | 19 | 29 | 24 | 26 | 26 | 26 |
| 38 | 29 | 17 | 26 | 24 | 25 | 24 | 26 |
| 39 | 27 | 15 | 23 | 21 | 23 | 23 | 24 |
| 40 | 26 | 14 | 22 | 20 | 22 | 21 | 22 |
| 41 | 25 | 13 | 21 | 19 | 21 | 21 | 22 |
| ≥42 | 27 | 13 | 21 | 18 | 20 | 21 | 22 |
