# Supplementary material for: Incremental cost of premature birth – a public health care payer perspective from Hungary
Source: BMC Health Serv Res. 2023 Jun 24;23:686. doi: 10.1186/s12913-023-09697-w (PMC10290341; doi:10.1186/s12913-023-09697-w)
Supplement: Supplementary file 3 — Additional file 3. [file 12913_2023_9697_MOESM3_ESM.docx]

**INCREMENTAL COST OF PREMATURE BIRTH – A PUBLIC HEALTH CARE PAYER PERSPECTIVE FROM HUNGARY**

S3. Pharmaceutical expenditure per capita (Euro) by gestational age (weeks) in different periods of life

| **Gest. age (weeks)** | **Periods of life** | | | | | | |
| --- | --- | --- | --- | --- | --- | --- | --- |
|  | **1^st^ half year** | **2^nd^ half year** | **2^nd^ year** | **3^rd^ year** | **4^th^ year** | **5^th^ year** | **6^th^ year** |
| 25 | 57 | 131 | 286 | 363 | 443 | 428 | 450 |
| 26 | 64 | 169 | 383 | 441 | 462 | 437 | 603 |
| 27 | 64 | 118 | 299 | 225 | 157 | 208 | 205 |
| 28 | 67 | 98 | 169 | 213 | 219 | 175 | 253 |
| 29 | 80 | 100 | 134 | 121 | 133 | 147 | 153 |
| 30 | 76 | 90 | 146 | 123 | 112 | 188 | 184 |
| 31 | 73 | 73 | 101 | 83 | 79 | 75 | 70 |
| 32 | 75 | 72 | 80 | 57 | 59 | 66 | 79 |
| 33 | 75 | 73 | 91 | 63 | 51 | 45 | 44 |
| 34 | 70 | 51 | 48 | 33 | 29 | 31 | 28 |
| 35 | 62 | 50 | 52 | 37 | 35 | 35 | 34 |
| 36 | 55 | 46 | 55 | 34 | 36 | 35 | 34 |
| 37 | 54 | 42 | 45 | 34 | 34 | 33 | 32 |
| 38 | 49 | 42 | 45 | 35 | 34 | 34 | 35 |
| 39 | 48 | 40 | 44 | 33 | 32 | 32 | 31 |
| 40 | 48 | 40 | 44 | 31 | 32 | 30 | 29 |
| 41 | 48 | 40 | 43 | 29 | 29 | 28 | 26 |
| ≥42 | 49 | 40 | 43 | 31 | 30 | 29 | 25 |
